# A Prospective Observational Study to Assess the Neutrophil-to-Lymphocyte Ratio (NLR) and C-reactive Protein-to-Albumin Ratio (CAR) in Predicting Morbidity and Mortality Among Patients Undergoing Emergency Abdominal Surgery

**DOI:** 10.7759/cureus.68369

**Published:** 2024-09-01

**Authors:** V Shrihari, Ramakanth Baloorkar, Shailesh S Kannur, Shivanagouda S Patil, Rohan D Gharpure

**Affiliations:** 1 Department of General Surgery, Shri B M Patil Medical College, Hospital and Research Centre, BLDE (Deemed to be University), Vijayapura, IND

**Keywords:** surgical site infection (ssi), emergency abdominal surgery, postoperative mortality, prognostic markers, c-reactive protein-to-albumin ratio, neutrophil to lymphocyte ratio (nlr), indian healthcare

## Abstract

Background

Emergency abdominal surgeries pose significant challenges, especially in the Indian population, due to comorbidities, delayed presentations, and limited resources. Accurately predicting morbidity and mortality is crucial for timely interventions and improved patient care. The neutrophil-to-lymphocyte ratio (NLR) and C-reactive protein-to-albumin ratio (CAR) have shown potential as prognostic markers, balancing inflammation and nutritional status.

Aim

The study aims to evaluate the predictive efficacy of NLR and CAR with regard to postoperative morbidity and mortality in patients undergoing emergency abdominal surgery, thereby contributing to better risk stratification and management strategies.

Patients and methods

A prospective observational study was conducted in a tertiary teaching hospital in northern Karnataka from August 2022 to June 2024, involving 102 patients undergoing emergency abdominal surgeries. The sample size (71) was calculated using G*Power software, targeting a 95% power with a 5% significance level. The inclusion criterion was patients aged over 18 years undergoing emergency abdominal surgeries; those who were immunocompromised, on steroid therapy, having malignancies, undergoing radiotherapy, or having chronic liver diseases were excluded from the study. Patients coming into the surgical inpatient department (IPD) with an acute abdomen requiring emergency abdominal surgeries as an emergency were preoperatively assessed using complete blood count (CBC), CRP, and serum albumin tests. NLR and CAR were evaluated preoperatively and at 24 and 48 hours postoperatively. The outcome measures included surgical site infection rates, hospital stay duration, and outcome in the form of recovery or death. SPSS version 20 was used for statistical analyses.

Results

The study included 102 patients whose mean age was 43.7 ± 18.9 years; 74 of the participants (72.5%) were male. The most common procedures were exploratory laparotomy (64 patients; 62.7%) and appendicectomy (32 patients; 31.4%). A significant increase in CAR levels was observed on postoperative days 1 and 2 compared to baseline (p < 0.05). Preoperative NLR ≥ 8 was significantly associated with higher mortality (65% vs. 50%, p < 0.01). Preoperative albumin > 3.2 g/dL was associated with better outcomes (recovery in 54 patients; 65.9%) compared to < 3.2 g/dL (15 patients; 75% mortality). This study showed that NLR and CAR are valuable predictors of postoperative outcomes, with CAR indicating the risk for surgical site infections (SSI) and NLR predicting mortality.

Conclusion

The preoperative NLR had a significant association with mortality among the patients. Hence the NLR can be a good marker for the worst outcome and CAR during the postoperative period can be considered as a marker to detect the risk of SSI. NLR and CAR are simple, inexpensive tests readily available from routine blood investigations. The utility of NLR and CAR as valuable prognostic markers in the perioperative assessment of patients undergoing emergency abdominal surgery could enhance the prediction of patient outcomes and guide more effective management strategies to improve patient outcomes in high-risk emergency abdominal surgery.

## Introduction

Emergency abdominal procedures are associated with several difficulties, particularly in the Indian population where results may be affected by a variety of comorbidities, delayed presentation, and inadequate resources. For precise intervention and better patient care, it is essential to predict morbidity and mortality in such circumstances [1]. Diagnosis involves history taking, physical examination, and based on the location of pain, although free air can lead to diffuse pain. Signs such as rebound tenderness, guarding, and absent bowel sounds may indicate peritonitis [2-4]. A more intense, localized pain indicates parietal peritoneum irritation, and referred pain to the right scapula indicates visceral peritoneum irritation [2-4].

Neutrophil-to-lymphocyte ratio (NLR) and C-reactive protein-to-albumin ratio (CAR) have emerged as potential prognostic markers, reflecting the balance between inflammatory status and nutritional status, respectively [5]. In the Indian context, where infectious diseases and malnutrition lead to a rising burden of non-communicable diseases, the use of these markers gains particular relevance [1].

The effectiveness of these ratios in predicting the results after an emergency abdominal surgery might be used to direct resource distribution and make clinical judgment. This research aims to assess the efficacy of NLR and CAR in predicting the morbidity and mortality among patients undergoing an emergency abdominal surgery.

## Materials and methods

A prospective observational study was conducted in a tertiary teaching hospital in northern Karnataka from August 2022 to June 2024. It involved 102 patients who were admitted with acute abdominal conditions and underwent emergency abdominal surgeries.

The sample size was calculated (71) using G*Power ver. 3.1.9.4 software, aiming for a 95% power to detect the differences in proportion with a 5% significance level, based on an anticipated inpatient mortality rate of 25% due to gastrointestinal perforation.

The inclusion criterion was adults above 18 years of age undergoing emergency abdominal surgeries, while exclusion criteria excluded immunocompromised patients, those on steroid therapy, individuals with malignancies, those undergoing radiotherapy, or those with chronic liver diseases.

The patients coming into the surgical inpatient department (IPD) and the Emergency for an acute abdomen requiring emergency abdominal surgeries were preoperatively assessed using complete blood count (CBC), CRP, and serum albumin tests, with subsequent evaluations of NLR and CAR at 24 and 48 hours postoperatively. The outcome measures included surgical site infection (SSI) rates, hospital stay duration, and outcome in the form of recovery or death. Statistical analysis involved data entry into Microsoft Excel (Microsoft, Redmond, WA), application of IBM SPSS Statistics, version 20 (IBM Corp., Armonk, NY), and a comparison of categorical variables by chi-square and Fisher's exact tests. For normally distributed continuous variables, independent sample t-tests were performed and non-normally distributed variables were compared by Mann-Whitney U tests. The results are presented as mean, standard deviation, counts, percentages, and graphical representations.

## Results

The present study included 102 patients with a mean age of 43.7±18.9 years who fulfilled the inclusion criterion. The majority of the participants were aged 26-40 years (32.4%) followed by those aged 41-60 years (29.4%) and 18-25 years (19.6%). Among the included patients, 74 (72.5%) were men and 28 (27.5%) were women.

**Figure 1:**
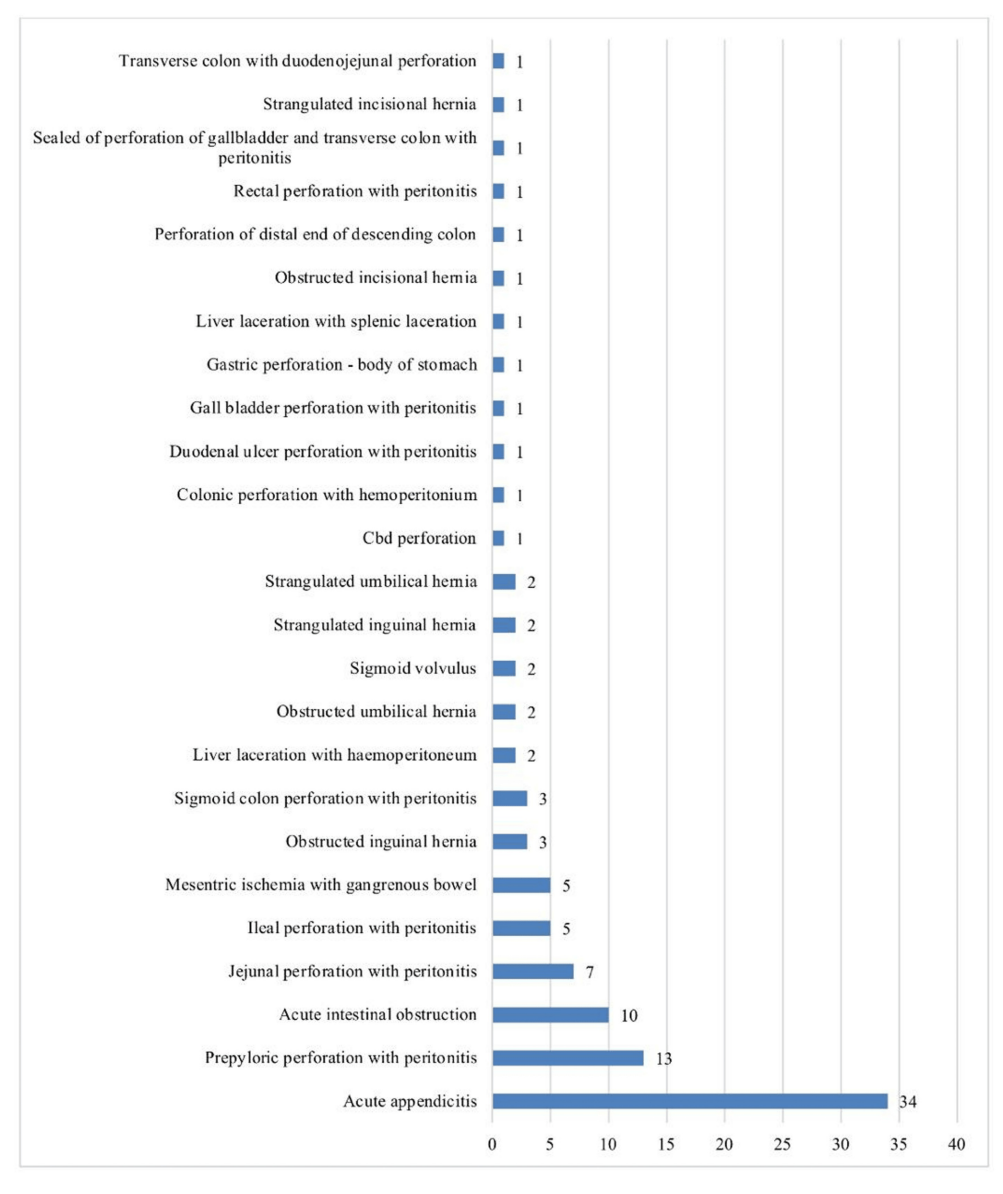
Distribution of the diagnosis of the patients.

Among the patients, the most common diagnosis at presentation was acute appendicitis in 34 patients (33.3%) followed by 13 patients (12.7%) with pre-pyloric perforation with peritonitis, 10 (9.8%) with acute intestinal obstruction, seven (6.9%) with jejunal perforation with peritonitis, two (2.0%) with obstructed inguinal hernia, two (2.0%) with strangulated umbilical hernia, two (2.0%) with strangulated inguinal hernia, and one (1.0%) with strangulated incisional hernia (Figure 1).

Among the procedures performed, 64 (62.7%) were exploratory laparotomy, 32 (31.4%) were appendicectomy, five (4.9%) were mesh hernioplasty for patients who presented with an obstructed hernia, and one (1%) was laparoscopic appendicectomy (Table 1).

**Table 1:** Distribution of procedures undergone by patients N: Number of patients.

Procedure	N	%
Exploratory laparotomy	64	62.7
Appendicectomy	32	31.4
Laparoscopic appendicectomy	1	1.0
Mesh hernioplasty	5	4.9

On comparing the change in the NLR level, we found a significant reduction in the level of NLR on postoperative days 1 and 2 compared to baseline levels (p<0.05). On comparing the change in CAR level, we found a significant increase in the level of CAR on postoperative days 1 and 2 compared to baseline levels (p <0.05) (Table 2).

**Table 2:** Comparison of the mean NLR and CAR among the patients NLR: neutrophil-to-lymphocyte ratio; CAR: C-reactive protein-to-albumin ratio; *: significant p-value Pre-op: Pre-operation; POD: postoperative day

		Mean	Standard Deviation	Paired P-value
NLR	Pre-op	11.61	11.98	0.01*
	POD-1	12.02	9.61	
NLR	Pre-op	11.61	11.98	0.01*
	POD-2	9.67	8.22	
CAR	Pre-op	0.0042	0.00528	0.01*
	POD-1	0.0072	0.00686	
CAR	Pre-op	0.0042	0.00528	0.01*
	POD-2	0.0071	0.01050	

An additional observation in the study is that the pre-op albumin level higher than 3.2 g/dl was observed in 59 (57.8%) patients. Surgical site infection was noted in 43 (42.2%) patients (Table 3).

**Table 3:** Patients with higher pre-op albumin values and contacting surgical site infection

		N	%
Pre-op albumin ≥3.2 g/dl	No	43	42.2
	Yes	59	57.8
Surgical site infection	No	13	12.7
	No	46	45.1
	Yes	43	42.2

Recovery was observed in 82 (80.4%) of the cases and 20 (19.6%) patients died. In the follow-up period, 15 (14.7%) patients succumbed within the initial 10 days of the postoperative period. The mean duration of hospital stay was 9.6 days (Table 4).

**Table 4:** Outcome for the patients at discharge and follow-up

		N	%
The outcome of management	Death	20	19.6
	Recovered	82	80.4
Follow-up days and number of patients who died	10	15	14.7
	30	1	1.0
	90	4	3.9

On comparison of the pre-op NLR with the outcome, there is a significantly higher incidence of death (13 patients; 65%) among the patients with NLR of more than 8 compared to patients who recovered (41 patients; 50%)

Also, the preoperative albumin with more than 3.2 g/dl led to better outcomes (54 patients; 65.9%) and an albumin level lower than 3.2 g/dl resulted in a higher incidence of death (15 patients; 75%) (Table 5).

**Table 5:** Comparison of the pre-op NLR and pre-op albumin with the outcome NLR: Neutrophil-to-lymphocyte ratio; * - significant p-value

		The outcome of management				Chi-square (p-value)
		Death		Recovered		
		N	%	N	%	
Pre-op NLR ≥8	No	7	35%	41	50.0%	1.45 (0.01)*
	Yes	13	65%	41	50.0%	
Pre-op albumin ≥3.2 g/dl	No	15	75%	28	34.1%	11.0 (0.01)*
	Yes	5	25%	54	65.9%	

**Figure 2:**
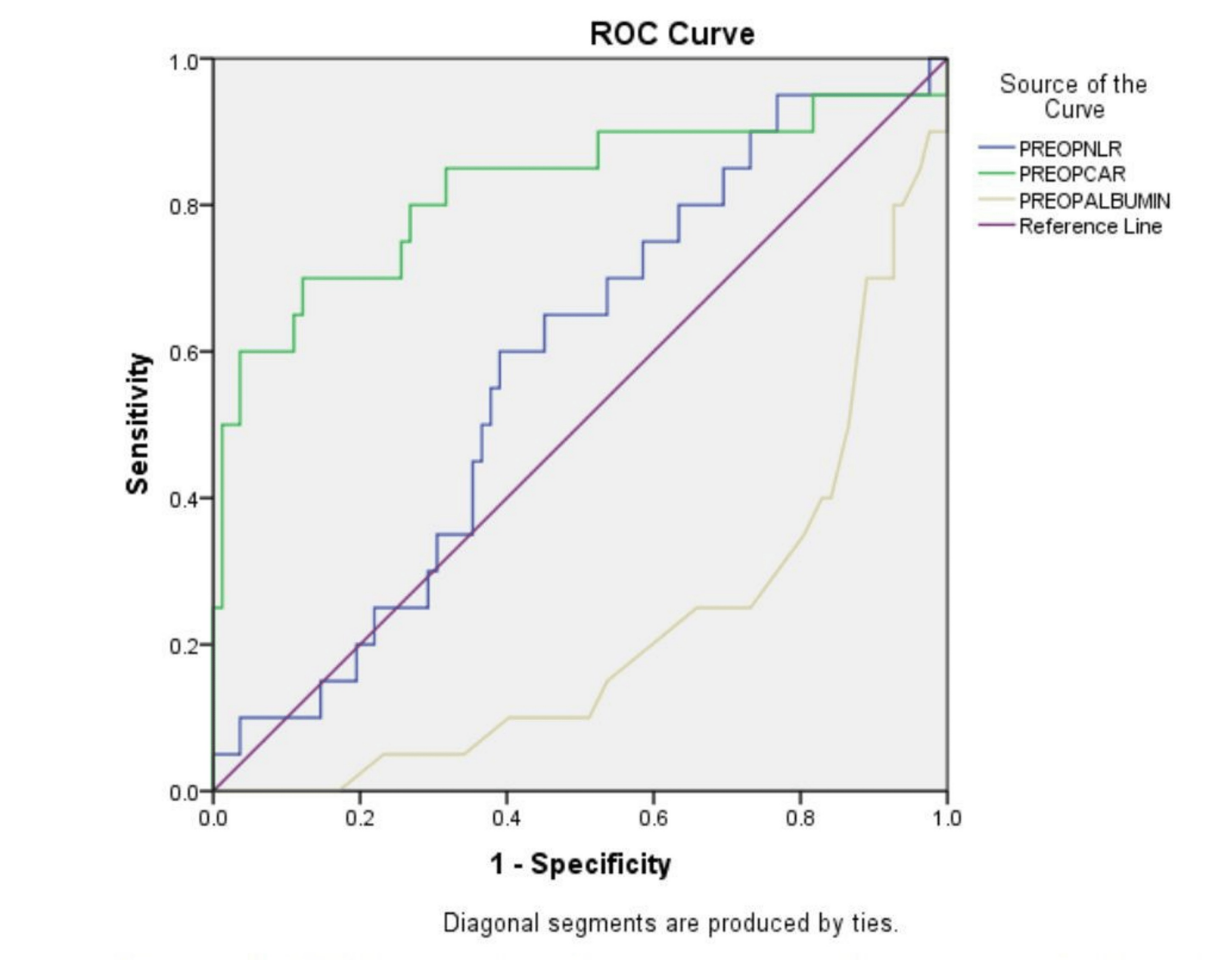
Receiver operating characteristic (ROC) curve showing the diagnostic characteristics of parameters

The receiver operating characteristic (ROC) analysis shows a negative relationship between preoperative albumin and the outcome and preoperative CAR and NLR show a significant positive correlation with the worst outcome for the patients (Figure 2 and Table 6).

**Table 6:** Area under the curve for parameters NLR: Neutrophil-to-lymphocyte ratio; CAR: C-reactive protein-to-albumin ratio.

Test Result Variable(s)	Area	Asymptotic Significance	Asymptotic 95% Confidence Interval	
			Lower Bound	Upper Bound
Pre-op NLR	0.578	0.273	0.450	0.709
Pre-op CAR	0.824	0.000	0.696	0.950
Pre-op albumin	0.219	0.000	0.110	0.326

## Discussion

NLR and CAR have become significant biomarkers in predicting morbidity and mortality among patients who undergo emergency abdominal surgery. These ratios reflect the body's inflammatory and nutritional status, respectively, providing valuable insights into a patient's physiological response to surgical stress and potential complications [6,7]. Elevated NLR and CAR levels have been associated with worse surgical outcomes, making them crucial tools for risk stratification and management in clinical practice. Comprehending and making use of these biomarkers can enhance preoperative assessment, guide postoperative care, and ultimately improve patient outcomes in emergency abdominal surgery settings.

In a study by Aquib [8], patients aged 20 or younger, or over 60 years, were in the majority, with a mean age of 39.6 ± 21.3 years. The male-to-female ratio was notably high at 3.6:1, with 78.3% male participants and 21.7% female participants [8]. Another study by Simpson et al. documented 84 years (range 80-96 years) as the median age [9]. The study by Makal and Yıldırım involved 326 patients with a mean age of 37 ± 11 years [10].

Acute intestinal perforation was the most frequent diagnosis (30%), followed by hollow viscus perforation (23.33%) in a study [8]. Intra-abdominal sepsis developed in 73.33% of patients post-surgery, with surgical site infection (SSI) being the most common (84.09%), followed by peritonitis (9.09%) [8].

In our study, the most common diagnosis at presentation was acute appendicitis (33.3%), followed by pre-pyloric perforation with peritonitis (12.7%), acute intestinal obstruction (9.8%), and jejunal perforation with peritonitis (6.9%). The procedures performed included exploratory laparotomy (62.7%), appendicectomy (31.4%), and mesh hernioplasty (4.9%).

A comparison of NLR levels revealed a significant reduction on postoperative days 1 and 2 compared to baseline levels (p < 0.05). Conversely, CAR levels showed a significant increase on postoperative days 1 and 2 compared to baseline levels (p < 0.05). Additionally, preoperative albumin levels above 3.2 g/dL were observed in 57.8% of the patients. SSIs were recorded in 42.2% of the patients. Aligning with the findings of the present study, Hançerlioğulları et al. also reported a notable reduction in NLR levels on postoperative days relative to the preoperative baseline levels [11].

A study by Buonacera et al. [1], highlighted NLR as an affordable and readily available biomarker that captures the interaction between acute and chronic inflammation and adaptive immunity. Despite the lack of defined threshold values for NLR, variations in its levels over time signal disturbances in immune function. NLR shows significant potential as a strong indicator of disease severity and mortality risk, though it requires adjustments for confounding factors and careful consideration of disease context, comorbidities, and treatment approaches [1].

A study by Donlon et al. [5] found that CRP levels exceeding 5 mg/dl and preoperative serum albumin levels below 32 g/dl were significant predictors of SSI. Interestingly, while the preoperative CRP/albumin ratio did not predict SSI, but the postoperative ratios at 24 and 48 hours were predictive. Furthermore, patients with SSI had a significantly longer median length of hospital stay compared to those who did not have the infection. In conclusion, while CRP and albumin levels are individually useful for preoperative SSI risk assessment, the CRP/albumin ratio is a valuable postoperative indicator at 24 and 48 hours [5].

In a study by Chen et al. [12], the CRP/albumin ratio closely correlates with inflammatory bowel disease (IBD) disease activity, demonstrating superior discriminative capacity compared to complete blood count (CBC) parameters [12].

In a study by Bora Makal and Yıldırım [10], the preoperative NLR emerged as a significant risk factor for the development of complications (OR = 1.943, p = 0.043). ROC analysis identified -0.78 and 1.25 mg/dL as cut-off values for CAR-1 and CAR-3, respectively, with corresponding area under the curve (AUC) values of 0.808 and 0.832. The study concluded that postoperative complications (POC) showed significant diagnostic value with CAR-3, followed by CAR-1, CRP-3, CRP-1, and platelet-to-lymphocyte ratio (PLR-3). Notably, preoperative NLR was the only risk factor identified for the development of POC [10].

In the present study, 80.4% of cases resulted in recovery, while 19.6% ended in death. During the follow-up period, 14.7% of the patients died within 10 days postoperatively. A comparison of preoperative NLR with outcomes revealed a significantly higher incidence of death (65%) among patients with an NLR higher than 8, compared to those who recovered (50%). Additionally, patients with preoperative albumin levels above 3.2 gm/dl had better outcomes (65.9%), whereas those with levels below 3.2 gm/dl had a higher incidence of death (75%).

In a study by Huang et al. [13], it was found that non-survivors exhibited a significantly higher mean NLR compared to survivors, demonstrating its superior prognostic value for predicting adverse outcomes in sepsis patients [13]. In a similar vein, a study by Tamai et al. [14] revealed that the CAR exhibited a significantly higher AUC compared to other markers like PLR, Gray platelet syndrome (GPS), NLR, and lymphocyte-to-monocyte ratio (LMR), except for the Prognostic Nutritional Index (PNI). The study identified optimal cut-off values for CAR as 0.106 and 44.894 for PNI. Patients with a CAR ≥ 0.106 experienced significantly poorer five-year recurrence-free survival (RFS), cancer-specific survival (CSS), and overall survival (OS) compared to those with a CAR < 0.106 [14].

In another study [15], a mortality rate of 49.4% was observed. Although no statistically significant differences were found in NLR and PLR values between survivors and non-survivors, the mean platelet volume (MPV) was significantly higher in the non-survivor group (p<0.004). The study concluded that higher MPV values were significantly associated with mortality following acute abdominal surgery, whereas NLR and PLR did not show a correlation with mortality [15].

On the other hand, research by Simpson et al. [9] showed no correlation between mortality and CAR. Finally, it was shown that in patients having emergency laparotomy due to visceral perforation, the preoperative NLR was associated with death; an NLR > 8 was associated with a worse prognosis. In contrast, CAR was not linked to death in patients older than 80 who had emergency laparotomies [9].

Strengths of the study

The study addresses a vital need for emergency abdominal surgeries for acute abdominal conditions, which are frequently fraught with complications due to comorbidities, delayed presentation, and inadequate resources. Concentrating on the predictive significance of NLR and CAR, the parameters of routine blood investigations, could help us modify the management strategies. The study found statistically significant relationships between higher NLR and CAR levels with poor surgical outcomes, such as surgical site infection and death.

Limitations of the study

This study's results need to be validated and strengthened by using a larger sample size. As ours was a single-center study, the results may not be generalizable to other healthcare settings with varying patient demographics and research availability. Some confounding factors like different surgical techniques and postoperative care protocols may influence the results and are not fully accounted for, even though the study excludes high-risk populations (e.g., immunocompromised patients, those on steroid therapy).

## Conclusions

Preoperative NLR had a significant association with mortality among the patients. NLR can be a good marker for the worst outcome and CAR during the postoperative period can be considered as a marker to detect the risk of SSI. NLR and CAR are simple, inexpensive tests readily available from routine blood investigations. The utility of NLR and CAR as valuable prognostic markers in the perioperative assessment of patients undergoing emergency abdominal surgery could enhance the prediction of patient outcomes and guide more effective management strategies to improve patient outcomes in high-risk emergency abdominal surgery.
